# The prevalence of traditional herbal medicine use among hypertensives living in South African communities

**DOI:** 10.1186/1472-6882-13-38

**Published:** 2013-02-18

**Authors:** Gail D Hughes, Oluwaseyi M Aboyade, Bobby L Clark, Thandi R Puoane

**Affiliations:** 1 South African Herbal Science and Medicine Institute, University of the Western Cape, Bellville 7535, South Africa; 2 Clark & Associates Statistical Consulting, Nolensville, TN, USA; 3 School of Public Health, University of the Western Cape, Bellville, 7535, South Africa

## Abstract

**Background:**

In South Africa, over 6 million people are hypertensive and the burden of disease shows that cardiovascular diseases (CVDs) are the leading cause of death among adults. Although treatments exist, few people comply or adhere to recommended treatment due to side effects or costs of the drugs, hence the reliance on alternative forms of treatment. Traditional herbal medicines (THM) are used for the management of hypertension but the prevalence of its use among hypertensive patients living in South African communities is not sufficiently known.

**Methods:**

This was a cross-sectional descriptive study to determine the prevalence of THM use for hypertension, among 135 purposefully selected South African participants of the Prospective Urban and Rural Epidemiological (PURE) study, who are THM users. Data on THM use were collected by way of face to face interviews using structured questionnaires administered by trained field workers. Standard descriptive measures were used to characterize the study sample and responses to the questionnaire. Chi-square test was used when making comparisons between groups.

**Results:**

There were 135 THM users, 21% of whom used THM to treat hypertension. Majority (82.1%) of the hypertensive THM users were females, only 29% were married or co-habitating, virtually all (96%) were unemployed and 86% were Christians. More than half (56%) of the respondents were aged between 55 and 64 years. THM was occasionally used (51.9%) as a combination of tea and other mixtures (63%) and prescribed by family/ friends/self-administered. There was a significant difference in the age, marital and employment status, as well as the form and frequency of THM use of hypertensive THM users compared to other THM users.

**Conclusions:**

The study gives an insight into the prevalence of THM use by hypertensive patients in selected South African communities. The practice of self-medication was also observed which raises concern regarding the safety of medications taken by the participants. Health care providers should however be more aware of THM use and counsel patients regarding the combination of prescribed treatment regimen and herbal medicines and the potential of herb-drug interactions.

## Background

Hypertension is defined as having a blood pressure ≥ 140/90 mmHg. It is a highly prevalent non-communicable disease estimated to affect about 800 million people globally [1]. Premature deaths resulting from hypertension was estimated at 7.1 million in 2003 with a global burden of disease of 4.5% [2]. In 2008, high blood pressure was the leading risk factor for deaths attributed to non-communicable diseases [3]. Hypertension is one of the chronic diseases partly attributed to behavioral factors alongside diabetes and obesity and is also an important risk factor for renal disease, blindness and for cardiovascular diseases (CVD) such as heart attacks, stroke and left ventricular hypertrophy. The prevalence of this disease is expected to increase by 24% and 80% in developed and developing countries respectively [4], and it has contributed to the present pandemic of CVDs particularly in developing countries.

Several decades ago, the prevalence of hypertension in some African countries was about 1% of the population [5]. However, globalization has resulted in health transitions and has revolutionized this disease from a rare medical condition to a major problem in sub-Saharan Africa [6]. About 20 million people in sub-Saharan Africa are hypertensive [6], prompting the African Union to recognize hypertension as one of the continent’s greatest health challenges after AIDS [7]. However, significant differences exist in the prevalence of hypertension between the urban and rural black populations in this region [8].

In South Africa, approximately every one in four persons between the ages of 15 and 64 years is hypertensive [9]. Based on race, black South Africans are more at risk of becoming hypertensive [10]. A study by Connor et al., showed that the age-standardised prevalence of hypertension was 59% for blacks, 55% for Indians and coloured and 50% for whites. Several studies have reported that more men than women are hypertensive in both urban and rural areas of South Africa [11,12]. However, latest statistics show that women are more at risk of developing hypertension (2.9 million) than men (2.6 million) [13].

The awareness of hypertension, its treatment and control thereof depends on the level of wealth of the patients [13]. Conventional drugs utilised in the management of hypertension include diuretics, brinerdin and ACE inhibitors. The latest hypertension guidelines for South Africa [14], however, provide a more comprehensive management protocol for hypertensive patients. These guidelines recommend that the target blood pressure for antihypertensive management be systolic <140 mmHg and diastolic <90 mmHg, with minimal or no drug side effects. However, stricter blood pressure control is required for patients with end-organ damage, co-existing risk factors and co-morbidity, e.g. diabetes mellitus. The reduction of blood pressure in the elderly and in those with severe hypertension should be achieved gradually over 1 month and co-existent risk factors should also be controlled. A more elaborate management protocol for hypertension in South Africa is described in the South African Hypertension Guidelines 2011 [14].

The inadequacy of the health care system in Africa and the economic impact of high cost of antihyper tensive drug treatment have made the use of THM common among hypertensive patients [1]. Several studies have reported the prevalence of complementary and alternative medicine use (CAM) in the treatment and management of hypertension; Morocco and Nigeria recorded 80% and 21% respectively [15,16]. However, very few publications exist about the prevalent of THM for hypertension. A study in India reported that 63.9% of hypertensive patients use THM [17]. In South Africa, surveys interviewing patients and traditional healers on the management and treatment of hypertension exists [18-22]. However, exact statistics on the prevalence of THM use by hypertensive patients in the country remain sparse. The study by Peltzer et al. showed that hypertensive patients consult traditional healers after visiting general practitioners in the Northern Province of South Africa [20]. Lokita also studied the reasons why hypertensive patients in Gauteng, South Africa consult traditional healers while using conventional drugs and found that some of the reasons for this include the positive attitude of the traditional healers compared to the health care providers, effectiveness of traditional medicine compared to conventional drugs with respect to side-effects and low cost of THM [23].

Conclusions from the study by Peltzer et al. recognised the relevant role which traditional healers play in the treatment and management of hypertension [20]. In the current study, we aimed to determine the prevalence of THM use among hypertensives in rural and urban communities in South Africa, specifically the Western Cape and Eastern Cape provinces.

## Methods

### Study design

A cross-sectional descriptive study was conducted utilizing a sample drawn from a population-based cohort study which involves adult males and females in South Africa. These subjects were recruited as part of the South African arm of a major prospective study – the Prospective Urban and Rural Epidemiological (PURE) Study [24], which recruited 2000 participants in total. In this study, a global cohort has been developed to investigate the impact of social and environmental transition on health and involves over 150, 000 adults initially aged 35 to 75 years from communities in 17 low-, middle- and high-income countries. A detailed description of the selection of this study population has been published elsewhere [24].

### Study setting

The current investigation was conducted in two communities of black South Africans namely: Langa, a township located in Cape Town in the Western Cape Province (urban), and Mount Frere, a small town located in the Eastern Cape Province (rural).

#### Sampling for PURE South Africa

The cohort was drawn to be representative of the adult population resident in both communities (Langa and Mount Frere) but also with mindfulness to the possibility of follow-up of participants. The communities were purposively selected on the basis of having a relatively stable (less migratory) black population thus allowing for feasibility of follow-up in a prospective cohort study. For the urban community (Langa), households were grouped into three development areas recognized administratively by the City of Cape Town and which mirror the socioeconomic status of the residents. A street map obtained from the City of Cape Town was used to randomly select streets in each of the 3 areas. Once a street was selected, a systematic sample of every second house was approached for possible inclusion in the study.

Household’s eligibility was based on the criteria that at least one member was between the age of 35 and 70 years, and that person intended to continue living in the current home for the next four years. All households with eligible individuals were approached by trained field workers for recruitment. All individuals who were “usual residents” were considered “household members” and eligible to be selected into the study. A “usual resident” was defined as one “who eats and sleeps in the household on most days of the week and in most weeks of the year and considered the household his/her primary place of habitation”.

For the rural community (Mount Frere), the absence of delineated streets precluded the possibility to follow the same sampling approach used for the urban township. A cluster sample of houses in the community was therefore undertaken according to the division of areas by the clan heads. All households within the clusters were included provided that there was a household member aged 30 to 70 years. The initial recruitment took place between April and August 2009 with close to 1000 participants recruited in both locations. A second phase recruitment took place between April and August 2010. The response rate was 85%. All the individuals who agreed to participate provided written informed consents

#### Sampling for the current study

The sampling frame for the current study was the 2000 participants who took part in the original South African PURE study. The sample size calculation performed using EPI-Info version 2007, showed that about 443 participants in total were required for the current study.

An administrative spread sheet used to capture participants’ information throughout the PURE follow-up period was used to randomly select a sample of 443 participants comprising of 222 participants from the rural site and 221 participants from the urban site. These participants were then interviewed to determine the prevalence of THM use. Only 135 were found to be using THM and thus constituted further analyses to determine THM use for hypertension and other conditions.

### Data collection

Data on the epidemiology of traditional medicine use for hypertension and other chronic conditions were collected from households and individuals in the study sample. Face to face interviews using structured questionnaires were conducted in these households between May and November 2010. The interviews were conducted by 8 trained data collectors in the preferred language of the respondent (English or Xhosa). Data were collected about the respondents’ demographic characteristics’ (age, sex, education, marital and employment status), clinical/medical history and traditional medicine usage (duration of use, condition for use, dosage, and form). The quality of data collected was maintained through the use of standardized protocols and centralized training. Excerpts from the questionnaire that asked about THM use include:

1. Are you using any herbal medicine?

2. Who prescribed the traditional medicine to you?

3. What condition are you treating with traditional medicine?

4. How often are you taking traditional medicine?

5. When did you start using traditional medicine?

### Data analysis

Descriptive statistics (e.g., frequencies and percentages) were used to characterize the study sample and responses to the questionnaire. Chi-square test was used when making comparisons between groups. All percent distributions were calculated based on non-missing values. No adjustments were made for missing data or multiple comparisons. A *p* value of <0.05 was considered statistically significant where applicable. All statistical analyses were performed using SAS 9.2.

### Ethical consideration

The study protocol was approved by the Senate Research Committee of the University of the Western Cape, South Africa.

## Results

The socio-demographic characteristics of the 135 respondents who reported using traditional herbal medicine are shown in Table 1. Approximately 72% were female, half (50%) were married or cohabitating, most were unemployed (55%), and an overwhelming 81% reported being Christian. Thirty-four per cent of the respondents were between the ages of 55 and 64, but only 24% were aged 44 or younger. Forty-one percent had no education or only primary education, while 54% had secondary education.

**Table 1:** Social and demographic characteristics of traditional medicine users (N=135)

**Variable**	**Frequency**	**Percent**
**Sex**		
Male	38	28.1%
Female	97	71.9%
**Age in years**		
≤ 44 years	31	24.2%
45-54 years	27	21.1%
55 - 64 years	43	33.6%
65+ years	27	21.1%
**Marital Status**		
Married / Cohabiting	68	50.4%
Divorced / Separated / Widowed	40	29.6%
Single	27	20.0%
Highest formal education completed*		
No education / Primary school	54	40.9%
Secondary school	71	53.8%
Vocational / Tertiary	7	5.3%
**Employment status**		
Working full time / Part time	22	16.3%
Unemployed / Looking for a job	74	54.8%
Retired / Other	39	28.9%
**Religion**		
Christian	110	81.5%
Traditional / No religion / Other	25	18.5%

*Missing Data: The 'education' variable has 3 missing cases.

Of the 135 participants who reported using traditional herbal medicine, only 28 (21%) used THM to treat hypertension (Table 2). None of the participants was younger than 44 years of age and very few were either married/co-habiting or had no education or only primary education.

**Table 2:** Social and demographic characteristics of hypertensive traditional medicine users (N= 28)

**Variable**	**Frequency**	**Percent**
**Sex**		
Male	5	17.9%
Female	23	82.1%
Age in years*		
< 44 years	0	0.0%
45-54 years	7	25.9%
55 -64 years	15	55.6%
65+ years	5	18.5%
**Marital Status**		
Married / Cohabitating	8	28.6%
Divorced / Separated / Widowed	13	46.2%
Single	7	25.0%
**Highest formal education completed**		
No education / Primary school	8	28.6%
Secondary school	19	67.9%
Vocational / Tertiary	1	3.6%
**Employment status**		
Working full time / Part time	1	3.6%
Unemployed / Looking for a job	13	46.2%
Retired / Other	14	50.0%
**Religion**		
Christian	24	85.7%
Traditional / No religion / Other	4	14.3%

*Missing data: The 'age' variable has 1 missing case.

A comparison between the social and demographic characteristics of hypertensive THM users and other THM users is presented in Table 3. Hypertensive THM users were significantly less likely to be either employed (4% versus 20%; p-value=0.009), under age 45 (0% versus 31%; p-value=0.003), and married or cohabitating (29% versus 56%; p-value=0.028), but more likely to be either female (82% versus 69%) and have a higher education (secondary, tertiary, or vocational school) than other THM users. However, there were no differences in religious affiliation between the users and non-users.

**Table 3:** Characteristics of hypertensive versus other traditional medicine users

	Hypertension*	**Other**	
**Variable**	**N (%)**	**N (%)**	**P-value**
**Sex**			
Male	5 (17.9)	33 (30.8)	
Female	23 (82.1)	74 (69.2)	0.174
**Age in years**			
< 44 years	0 (0.0)	31 (30.7)	
45-54 years	7 (25.9)	20 (19.8)	
55 -64 years	15 (55.6)	28 (27.7)	
65+ years	5 (18.5)	22 (21.8)	0.003
**Marital Status**			
Married /Cohabiting	8 (28.6)	60 (56.1)	
Divorced / Separated /Widowed	13 (465.4)	27 (25.2)	
Single	7 (25.0)	20 (18.7)	0.028
**Highest formal education completed**			
No education /Primary school	8 (28.6)	46 (44.2)	
Secondary school	19 (67.9)	52 (50.0)	
Vocational /Tertiary	1 (3.6)	6 (5.8)	0.243
**Employment status**			
Working full time /Part time	1 (3.6)	21 (19.6)	
Unemployed /Looking for a job	13 (46.4)	61 (57.0)	
Retired /Other	14 (50.0)	25 (23.4)	0.009
**Religion**			
Christian	24 (85.7)	86 (80.4)	
Traditional /No religion /Other	4 (14.3)	21 (19.6)	0.517
**Form of TM**			
Tea	3 (11.1)	33 (33.3)	
Powder	1 (3.7)	13 (13.1)	
Tea and other mixture	17 (63.0)	14 (42.4)	
Other	6 (22.2)	11 (11.1)	0.027
**Frequency of use**			
1-3 times per day	13 (48.1)	28 (27.5)	
1-3 times per week	0 (0.0)	14 (13.7)	
Occasionally	14 (51.9)	60 (58.8)	0.034
**Prescriber of TM**			
Traditional Healer / Spiritualist /Diviner	3 (13.6)	34 (34.7)	
Family Member / Friend / Self-administered	15 (68.2)	39 (39.8)	
Different prescribers	4 (18.2)	25 (25.5)	0.045
**Length of time using TM**			
<= 1year	3 (10.7)	10 (9.9)	
> 1year	3 (10.7)	20 (19.8)	
Several years ago	20 (71.4)	58 (57.4)	
Unsure	2 (7.1)	13 (12.9)	0.501

*Respondents who used TM for hypertension may also have used TM for other conditions as well. These respondents are represented in the 'Hypertension' column.

Table 3 presents the usage characteristics of hypertensive THM users versus other THM users. Other THM users were three times more likely to take THM in the form of tea and about four times likely to take THM as a powder compared to hypertensive THM users. However, hypertensive THM users were more likely to take THM as tea with other mixtures than other users (63% versus 42%). The differences in THM usage forms were statistically significant with a *p*-value of 0.027. Hypertensive users took THM more frequently than other THM users. Forty-eight percent of hypertensive users took THM 1 to 3 times per day compared to only 27.5% of other THM users. Other THM users were more likely to take THM occasionally (59% versus 52%).

Prescribers of THM to other THM users were more than twice likely to be traditional healers, spiritualist, or diviners. This is in contrast to hypertensive THM users who were more likely to use THM based on family members and friends’ prescription, or to self-prescribed THM. These differences in THM prescribers were statistically significant with a p-value of 0.045.

Lastly, the length of time respondents had been using THM was determined. Hypertensive THM users were more likely to have used THM for several years compared to other THM users (71% versus 57%).

## Discussion

The purpose of the study was to determine the prevalence of THM use by hypertensives in a convenient sample of the PURE cohort. The study showed that of the 135 participants who indicated using THM, 21% used THM in the treatment and management of hypertension. This estimate is similar to that observed in a study in Nigeria (21%) [16]. However, it is lower than the estimates reported in other countries such as Morocco (80%) [15] and India (63.9%) [17]. Also, a community-based study in Nigeria reported an estimate of 29% of the participants who employed CAM to manage hypertension [1]. Interestingly, another study in Nigeria revealed that majority of hypertensive patients considered traditional medicine as the only viable cure for hypertension [16]. Although the current study is limited in terms of geographical representation, it begins to show how South Africa, particularly within the back communities, compares to other countries in terms of THM use among patients with hypertension.

The use of THM by the study participants for other varied illnesses ranged from common cough and cold to respiratory problems and diabetes. Also, some respondents indicated using THM for general well-being and maintenance of health. This, to a certain degree, reflects the role of THM in managing certain disease conditions over and above the western medical practices in South Africa. In fact, it mirrors the patterns of THM use in other regions of African and the world. For example, THM accounts for 65% of all health care in India, 71% in Chile, 40% in China and over 80% in most of the African countries [25], especially in their rural communities where traditional herbal medicine is often the most culturally acceptable choice of health care [26].

Our study revealed that age, marital and employment statuses were significantly associated with THM use for hypertension, a pattern that has also been reported in another study [1]. However, other studies that examined similar demographics did not show any association between age, marital and employment status and the use of THM [27,28]. The association observed between employment status and the use of THM for hypertension is an indication of the health seeking behaviour of the respondents. Our study showed that THM was mostly used for treating hypertension by either the unemployed or the retirees. The rising cost of for managing chronic diseases within the formal healthcare system in South African could be one of the reasons why the retired and unemployed participants in the current study chose to use THM for hypertension [1].

The prescription of THM for hypertension in this study was influenced by family, friends or self-administration. A similar report has shown that users of herbal medicinal products in the United States do not seek professional advice in selecting herbal medicines, but rather rely on friends’ or relatives’ recommendations [29]. The use of THM for various illnesses has been strongly associated with family influence and cultural traditions. Family expectations of receiving treatment from traditional healers and cultural beliefs are some of the reasons for continuous dependence on THM [30,31]. The practice of self-diagnosis and medication of traditional medicine knowledge is wide spread in urban areas of developing countries. Cocks and Dold, observed that all age groups and social categories of people who took part in their study medicated for both themselves and their family members [32].

The wide spread of traditional health shops offering a wide choice of medicines for diverse ailments and problems in various cities in South Africa is a source of concern with regards to the efficacy and ultimately the safety of such herbal products. The apparent lack of THM prescriptions by trained personnel for hypertension in this study is indicative of indigenous knowledge for healing purposes being handed down from one generation to another within household settings. In a review of the use and practice of traditional/complementary medicine in South Africa, Peltzer, observed a decrease in the use of traditional healers from between 3.6-12.7% to 0.1% in South Africa in the last 13 years [25]. However, in our study, we observed that the participants who use THM for other health conditions apart from hypertension were more likely to obtain their prescription from traditional healers.

While half (51%) of the participants indicated occasional THM use, 48% indicated 1–3 times a day use. The frequency of use of THM and the length of time herbal remedies have been used by the participants are indicative of a heavy dependence on THM not only in managing hypertension but also in treating it. This however poses a threat to the health of the participants as the possibility of herb-drug interaction is high among those who use dual therapy. Co-administration of herbal remedies with cardiovascular medications with narrow therapeutic index (warfarin and digoxin) has been found to potentiate or reduce the effect of these medications [33]. Health care professionals should be aware of increased THM usage amongst their patients and thus be able to give proper medical advice with regards to potential adverse effects or possible herb-drug interactions.

The study had several limitations that need to be acknowledged. Firstly, the small sample size affected the external validity of the study. Thus this study cannot be generalized to a broader population. Secondly both the estimates of the prevalence of THM use and hypertension were based on self-reported behaviour, and we speculate some degree of under-reporting which is a possibility in survey of this nature [41]. Also, the types/levels of hypertension and the length of time since diagnosis were not assessed in this study. However, this study still provides insight into the use of THM among participants self-reporting hypertension.

## Conclusions

This study has identified the prevalence of THM use by hypertensives living in selected South African communities. The practice of self-medication for hypertension treatment by traditional herbal medicine users was observed. Also, the length of time during which THM has been used by the participants raises questions about adherence to prescription medication. Health care workers should therefore be aware of, and inquire about THM use among hypertensive patients, in order to advise patients on the potential of herb-drug interaction and possible adverse effects. Future investigations should explore long-term use of THM within the cohort, response to treatment including changing health status while simultaneously taking THM and conventional medicines, and carefully studying the timing of initiation of THMs.

## Abbreviations

ACE: Angiotensin converting enzymes; CAM: Complementary and alternative medicine; CVD: Cardiovascular diseases; PURE: Prospective Urban and Rural Epidemiology; THM: Traditional herbal medicine; WHO: World Health Organization.

## Competing interest

The authors declare they have no competing interest.

## Authors’ contributions

GH and TP conceptualized the study, contributed to writing and finalized the manuscript. OA drafted the manuscript and BC did the statistical analysis. All authors read and approved the final manuscript.

## Pre-publication history

The pre-publication history for this paper can be accessed here:

http://www.biomedcentral.com/1472-6882/13/38/prepub
